# Endogenous IL-6 of mesenchymal stem cell improves behavioral outcome of hypoxic-ischemic brain damage neonatal rats by supressing apoptosis in astrocyte

**DOI:** 10.1038/srep18587

**Published:** 2016-01-14

**Authors:** Yan Gu, Mulan He, Xiaoqin Zhou, Jinngjing Liu, Nali Hou, Tan Bin, Yun Zhang, Tingyu Li, Jie Chen

**Affiliations:** 1 Children Nutrition Research Center, Children's Hospital of Chongqing Medical University, Chongqing 400014, China; 2 Ministry of Education Key Laboratory of Child Development and Disorders, Children's Hospital of Chongqing Medical University, Chongqing 400014, China; 3 Chongqing Stem Cell Therapy Engineering Technical Center, Children's Hospital of Chongqing Medical University, Chongqing 400014, China; 4 Chongqing Key Laboratory of Translational Medical Research in Cognitive Development and Learning and Memory Disorders, Chongqing 400014, China

## Abstract

Mesenchymal stem cell (MSC) transplantation reduces the neurological impairment caused by hypoxic-ischemic brain damage (HIBD) via immunomodulation. In the current study, we found that MSC transplantation improved learning and memory function and enhanced long-term potentiation in neonatal rats subjected to HIBD and the amount of IL-6 released from MSCs was far greater than that of other cytokines. However, the neuroprotective effect of MSCs infected with siIL-6-transduced recombinant lentivirus (siIL-6 MSCs) was significantly weakened in the behavioural tests and electrophysiological analysis. Meanwhile, the hippocampal IL-6 levels were decreased following siIL-6 MSC transplantation. *In vitro*, the levels of IL-6 release and the levels of IL-6R and STAT3 expression were increased in both primary neurons and astrocytes subjected to oxygen and glucose deprivation (OGD) following MSCs co-culture. The anti-apoptotic protein Bcl-2 was upregulated and the pro-apoptotic protein Bax was downregulated in OGD-injured astrocytes co-cultured with MSCs. However, the siIL-6 MSCs suppressed ratio of Bcl-2/Bax in the injured astrocytes and induced apoptosis number of the injured astrocytes. Taken together, these data suggest that the neuroprotective effect of MSC transplantation in neonatal HIBD rats is partly mediated by IL-6 to enhance anti-apoptosis of injured astrocytes via the IL-6/STAT3 signaling pathway.

Neonatal hypoxic-ischemic brain damage (HIBD) is a leading cause of mortality and neurological disability in infants and young children and occurs in 1 to 6 of every 1,000 live term births. The treatment and care of the sequelae of HIBD require extensive resources. However, even with the best care, there is often little improvement in the overall ability of these children. Consequently, HIBD is a major public health issue globally with long-term effects on the family, health care system, and society^1^.

Stem cells have attracted attention from scientists due to their potential to induce tissue repair and regeneration. Based on ethical issues, safety concerns, sources and tumourigenicity, mesenchymal stem cells (MSCs) derived from bone marrow are considered an appropriate choice for HIBD treatment^2^. Numerous studies have demonstrated that MSC transplantation is neuroprotective for HIBD. A majority of studies have supported the immunomodulatory function of MSCs in the injured microenvironment of HIBD because MSCs can release various factors to regulate local immune responses or support the survival of vulnerable neurons^3^,^4^,^5^,^6^,^7^,^8^,^9^. We previously demonstrated that MSCs exert neuroprotective effects on H_2_O_2_-exposed PC12 cells partly via IL-6 and IL-10 cytokine production^10^.

IL-6 is a pleiotropic and multifunctional cytokine involved in modulating various physiological events, such as cell proliferation, differentiation, survival, and apoptosis^11^. Generally, the biological functions of IL-6 are achieved via the Janus-associated kinase (JAK)–signal transducer and activator of transcription (STAT) pathway, the Ras–Raf–mitogen-activated protein kinase (MAPK) pathway and the PI3K/Akt pathway^12^,^13^. In the central nervous system (CNS), the IL-6 levels are low under normal conditions but are significantly elevated under disease conditions. Both detrimental and beneficial functions of this cytokine have been reported^14^,^15^, but the exact role of this cytokine following MSC transplantation has not been established.

We recently observed that MSCs release extremely high levels of IL-6 compared with other cytokines. Because MSC transplantation improves behavioural outcomes in HIBD rats^3^,^4^,^5^,^6^, we speculated that IL-6 may exert a neuroprotective effect during MSC treatment for HIBD. Therefore, we conducted the following series of experiments. First, we treated neonatal HIBD rats with MSCs infected with siIL-6-transduced lentivirus (siIL-6 MSCs) to verify the biological role of IL-6 in promoting the recovery of learning and memory deficits in rats subjected to HIBD. Second, MSCs were separately co-cultured with primary neurons or astrocytes treated by oxygen-glucose deprivation (OGD) to identify the target cells of IL-6 released by MSCs. Finally, siIL-6 MSCs were used to explore the signalling pathways by which endogenous IL-6 released from MSCs improves learning and memory function following HIBD. The results of this study may provide supporting information for the clinical application of MSCs.

## Results

### MSC transplantation alleviates cognitive impairment in rats subjected to HIBD

**Figure 1:**
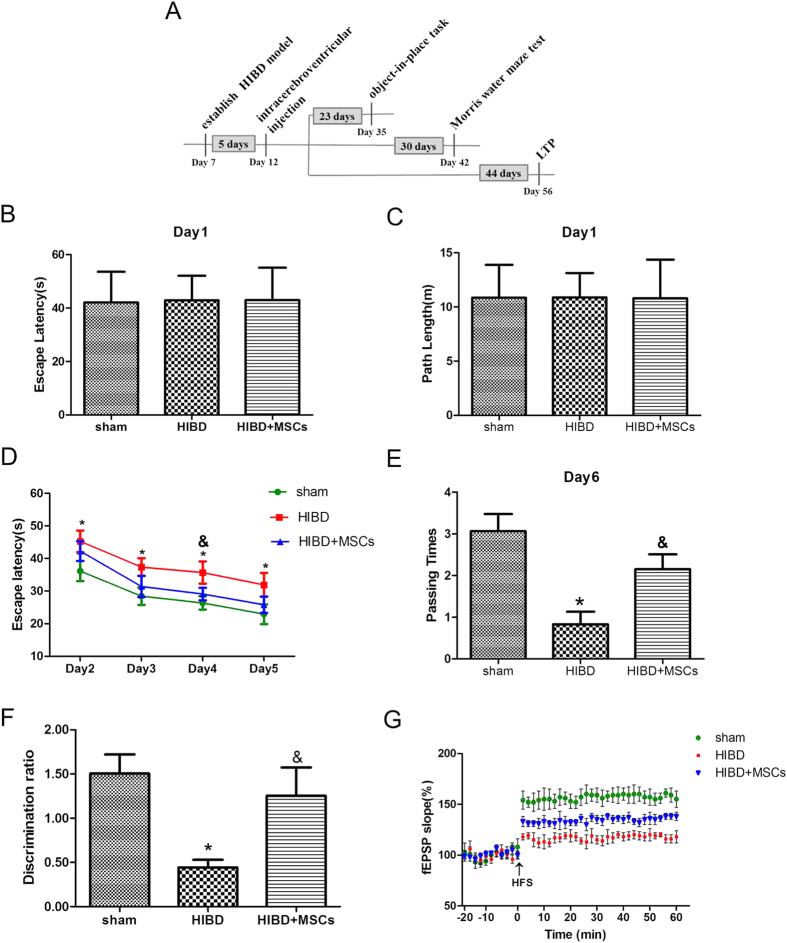
MSC transplantation restores learning and memory function in neonatal HIBD rats. (**A**) Diagram illustrating the experimental protocols of treatments and tests for rats in the sham, HIBD and HIBD + MSCs groups. (**B,C**) The escape latencies and path lengths to reach the visible platform on the first day of the Morris water maze test for the sham, HIBD and HIBD + MSCs rats. (**D**) The escape latencies of each group to locate the visible platform from the 2nd to the 5th day in the Morris water maze test. (**E**) The number of passes through the former platform region of each group on the final day of the Morris water maze test. n = 15. (**F**) The discrimination ratio of the exploration time during the test phase of the object-in-place task among the three groups. n = 15. (**G**) The mean fEPSP slope of the three groups. n = 6. The results are presented as the mean ± SEM. *P < 0.05 vs. the sham group, ^&^*P* < 0.05 vs. the HIBD group.

Behavioural deficits, particularly impairment in learning and memory functions, are the major sequelae of HIBD^16^. To identify the effects of MSC transplantation on memory damage in rats subjected to HIBD, behavioural tests and electrophysiological recordings were conducted. As shown in Fig. 1B,C, on the first day of the Morris water maze test, the escape latency and path length required to locate the platform did not differ significantly among the Sham, HIBD and HIBD + MSCs groups. This result indicated that neither HIBD nor MSC transplantation impaired the rats’ motility or vision. During the four-day training period for the hidden platform test, the escape latency decreased progressively in all groups. However, the rats in the HIBD group required more time to locate the platform than the rats in the other two groups, although the escape latency of the HIBD + MSCs group was longer than that of the sham group (Fig. 1D). In the spatial probe test, the HIBD group passed through the original platform region least among the three groups. The number of passes was greater for the HIBD + MSCs group than the HIBD group (Fig. 1E). In the object-in-place memory task, the rats in the HIBD group spent less time exploring the objects in novel positions, whereas the HIBD + MSCs rats preferentially responded to the displaced objects (Fig. 1F). The field excitatory postsynaptic potentials (fEPSPs) in the hippocampal CA1 region of brain slices from each group were recorded to examine the effects of MSC transplantation on hippocampal synaptic plasticity. The basal levels of fEPSPs in the three groups were recorded 20 min before and 60 min after high-frequency stimulation (HFS). After HFS was applied, the mean increase in the slope of the fEPSPs in the HIBD group (117.1% ± 4.6%) was significantly less than that in the sham group (156.3% ± 7.3%). However, the slope of the fEPSPs in the HIBD + MSCs group (134.2% ± 4.3%; Fig. 1G) was more than that in the HIBD group despite that the difference was with no significant. These results demonstrated that MSC transplantation alleviated the memory impairment of rats subjected to HIBD.

### MSCs release high level of IL-6 and infection of siIL-6-transduced recombinant lentivirus decreases the IL-6 release of MSCs

**Figure 2:**
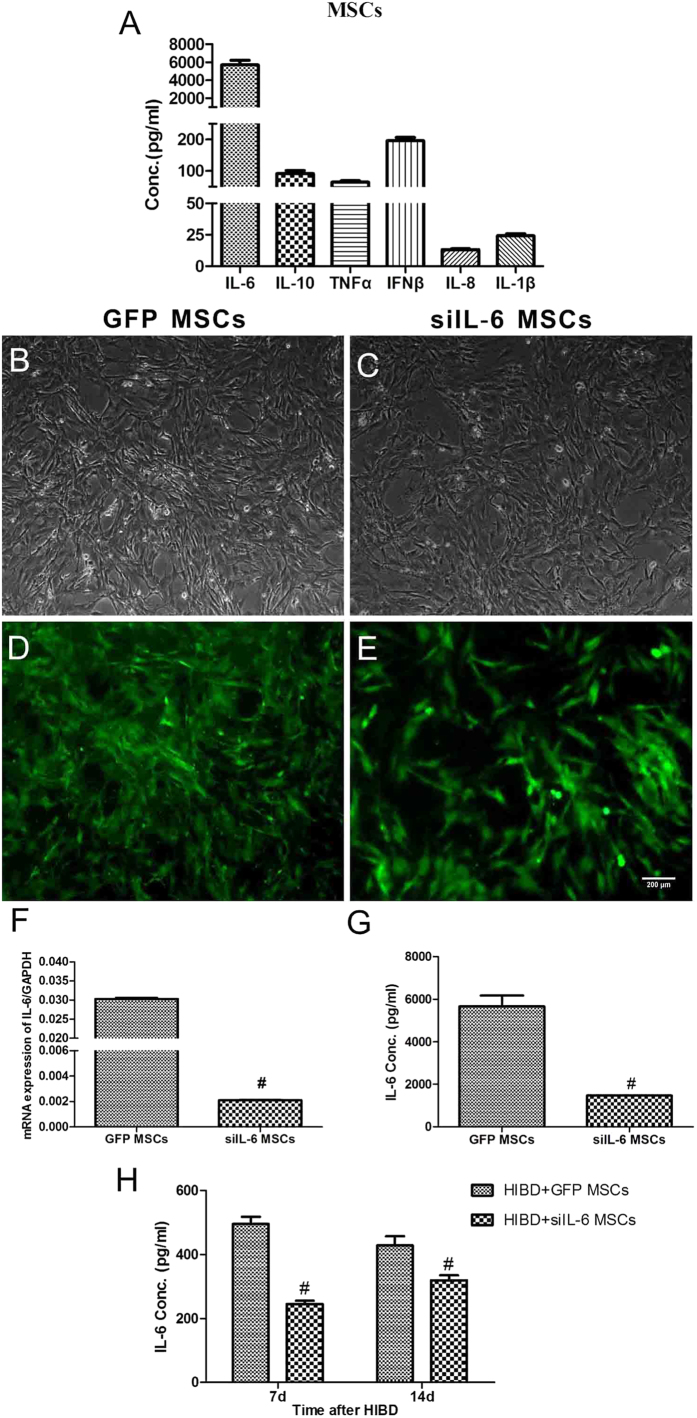
High level of IL-6 secreted by MSCs and was reduced in MSCs infected with siIL-6-transduced lentivirus. (**A**) The concentrations of the cytokines IL-6, IL-1β, IL-8, IL-10, TNFα and IFNβ in the culture medium of primary MSCs. n = 4. (**B,C**) Primary MSCs infected with lentivirus transduced with an empty vector that expressed GFP or infected with siIL-6-transduced recombinant lentivirus. (**D,E**) The efficiency of lentivirus infection in MSCs between the siIL-6-transduced recombinant lentivirus and its control. (**F,G**) The levels of IL-6 mRNA expression and protein release by GFP MSCs and siIL-6 MSCs. n = 3. Scale bar = 200 μm. (**H**) The concentration of IL-6 in the hippocampus of HIBD + siIL-6 MSCs and HIBD + GFP MSCs rats at 7 and 14 days after HIBD. n = 5. The results are presented as the mean ± SEM. ^#^*P* < 0.05 vs. the GFP MSCs group.

Previous studies have attributed the neuroprotective effect of MSCs more to their immunomodulatory functions than other mechanisms^17^,^18^. Therefore, we measured the levels of cytokines released by MSCs. The IL-6 levels were extremely high compared with those of IL-1β, IL-8, IL-10, TNFα and IFNβ (Fig. 2A), indicating that IL-6 might be involved in the recovery process after MSC transplantation in rats subjected to HIBD. To further verify the effect of IL-6 released by MSCs, we constructed a siIL-6-transduced recombinant lentivirus and used it to infect the MSCs. The lentivirus infected MSCs with high efficiency (Fig. 2B–E) and effectively suppressed the expression and release of IL-6 in MSCs (Fig. 2F,G). The IL-6 levels at 7d and 14d after HIBD were significantly lower in the brains of neonatal rats transplanted with siIL-6 MSCs than in those transplanted with GFP-transduced lentivirus-infected MSCs (GFP MSCs) (Fig. 2H).

### The neuroprotective effect of MSCs is weakened after infection with siIL-6-transduced recombinant lentivirus

**Figure 3:**
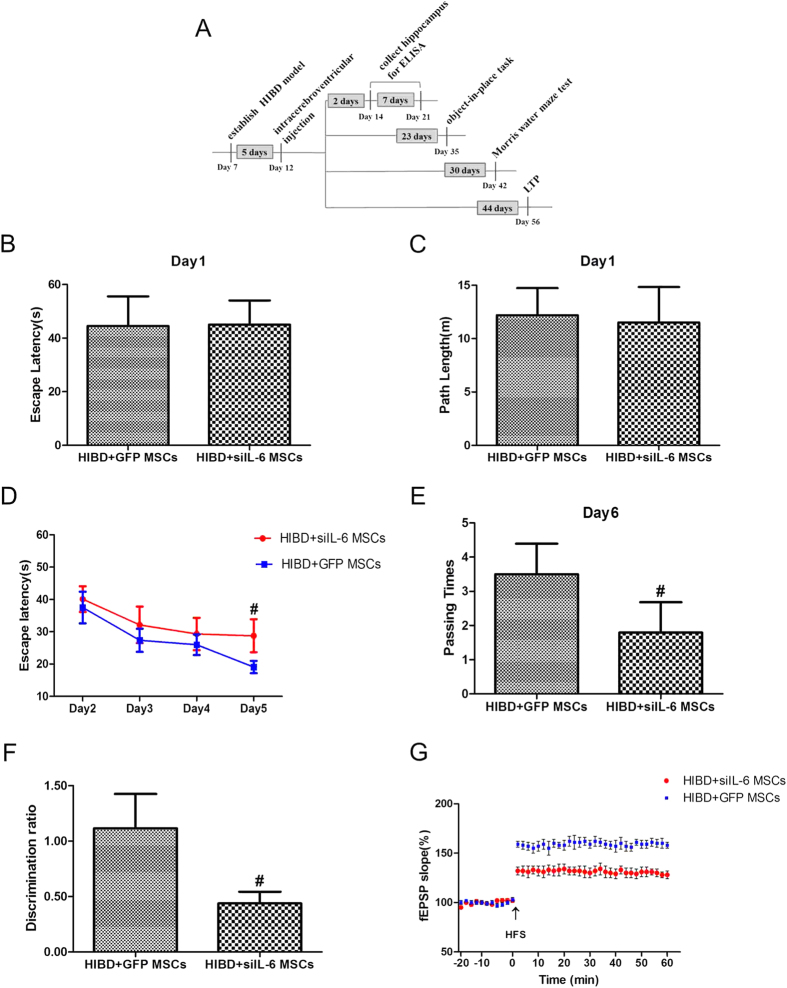
The neuroprotective effect of MSCs is weakened when IL-6 expression is silenced. (**A**) Diagram illustrating the experimental protocols of the treatments and tests for rats in the HIBD + siIL-6 MSCs group and HIBD + GFP MSCs group. (**B,C**) The escape latencies and path lengths to reach the visible platform on the first day of the Morris water maze test for rats in the two groups. n = 15. (**D**) The escape latencies of each group to locate the visible platform from the 2nd to 5th day in the Morris water maze test. (**E**) The passing times through the former platform region of the two groups on the final day of the Morris water maze test. (**F**) The discrimination ratio of the exploration time during the test phase of the object-in-place task between the HIBD + siIL-6 MSCs and HIBD + GFP MSCs groups. n = 15. (**G**) The standardised fEPSP slope of the two groups. n = 6. The results are presented as the mean ± SEM. ^#^*P* < 0.05 vs. the HIBD + siIL-6 MSCs group.

To determine whether IL-6 plays a vital role in the neuroprotective effect of MSCs, a stable MSC line infected with siIL-6-transduced recombinant lentivirus was constructed and transplanted into the brains of neonatal HIBD rats. On the first day, the escape latency and path length in the Morris water maze test were identical in the HIBD + GFP MSCs group and HIBD + siIL-6 MSCs group (Fig. 3B,C), demonstrating that there were no differences in the motility and vision of rats between the two groups. However, during the directional navigation training, the escape latency of the HIBD + siIL-6 MSCs group was longer than that of the HIBD + GFP MSCs group (Fig. 3D). On the final day of testing, rats in the HIBD + siIL-6 MSCs group passed fewer times through the original platform region compared with rats in the HIBD + GFP MSCs group (Fig. 3E). In the object-in-place memory test, rats in the siIL-6 MSCs group displayed a significantly lower discrimination score than the control group (Fig. 3F). Similarly, the increase in the fEPSPs of the HIBD + siIL-6 MSCs group (131.2% ± 4.8%) was significantly smaller than that of the HIBD + GFP MSCs group (160% ± 4.5%) (Fig. 3G). The above results suggest that the neuroprotective effect of MSCs is partly mediated by endogenous IL-6.

### Co-culturing OGD-injured neurons with MSCs activates the IL-6/STAT3 signaling pathway but does not affect the ratio of Bcl-2/Bax

**Figure 4:**
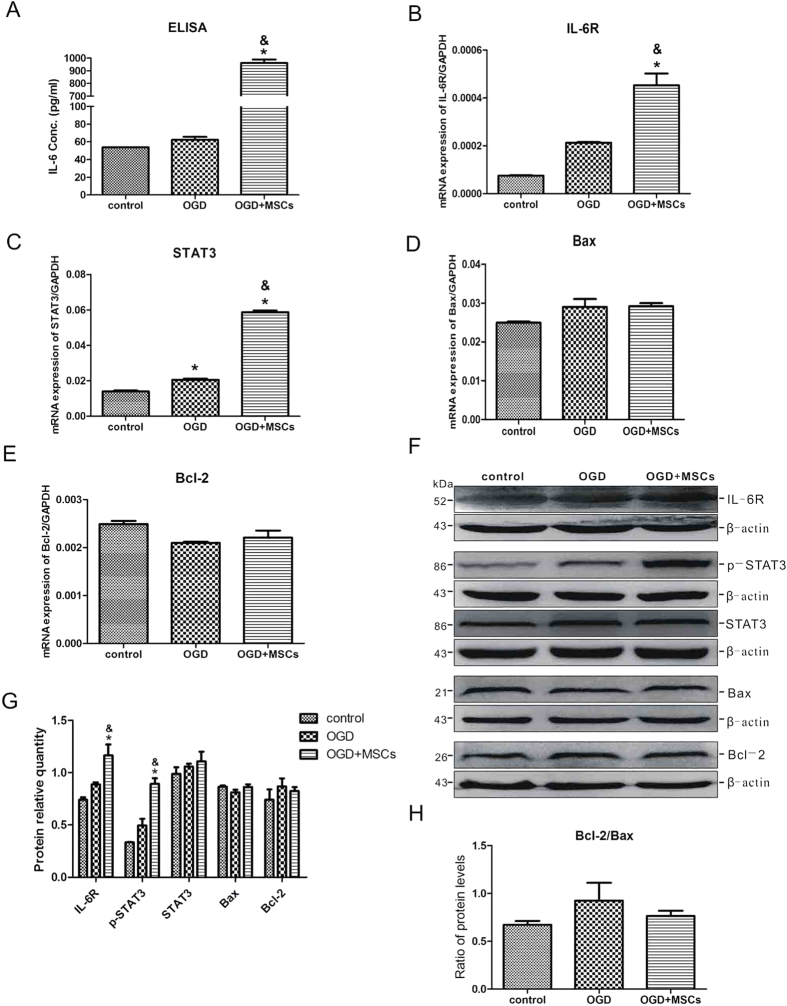
MSC co-culture activates the IL-6/STAT3 signalling pathway in OGD-injured neurons but has no effect on the ratio of Bcl-2/Bax. (**A**) The concentration of IL-6 released in the culture medium of normal neurons and OGD-injured neurons with or without MSC co-culture as determined by ELISA. n = 4. (**B–E**) The mRNA expression levels of IL-6R, STAT3, Bax and Bcl-2 in the three groups. n = 4. (**F**) Representative western blot of IL-6R, p-STAT3, STAT3, Bax and Bcl-2 expression in neurons from the control, OGD and OGD + MSCs groups. n = 4. (**G**) The quantifications of WB signal in F. (**H**) The ratio between Bcl-2 and Bax protein levels in the three groups. The results are presented as the mean ± SEM. *P < 0.05 vs. the control group, ^&^*P* < 0.05 vs. the OGD group.

To confirm the neuroprotective effect of endogenous IL-6 from MSCs and its possible mechanism, an *in vitro* OGD model with neurons was used to simulate *in vivo* HIBD. Although IL-6 functions in brain damage through different signalling pathways, several studies have revealed that the IL-6/STAT3 signaling pathway plays a major role^19^,^20^. We therefore detected the key factors in the IL-6/STAT3 signaling pathway, including IL-6, IL-6R, STAT3, Bax and Bcl-2. As shown in Fig. 4A, OGD injury did not influence the expression of IL-6 conspicuously, whereas OGD-injured neurons co-cultured with MSCs exhibited significantly increased release of IL-6 in neurons. IL-6R and STAT3 expression were highly consistent with the changes in IL-6 at the mRNA level (Fig. 4B,C). However, neither OGD injury nor MSC co-culture affected the levels of Bax and Bcl-2 mRNA expression (Fig. 4D,E). The protein expression levels of IL-6R, p-STAT3, Bax and Bcl-2 were consistent with their mRNA expression levels, except that the protein expression level of STAT3 was not influenced by OGD or the co-culture treatments (Fig. 4F,G). The ratio of the Bcl-2 and Bax protein levels did not differ significantly among the treatments (Fig. 4G,H). These results reveal that endogenous IL-6 release from MSCs activated IL-6R and STAT3 but had no effect on the downstream factors Bax and Bcl-2.

**Figure 5:**
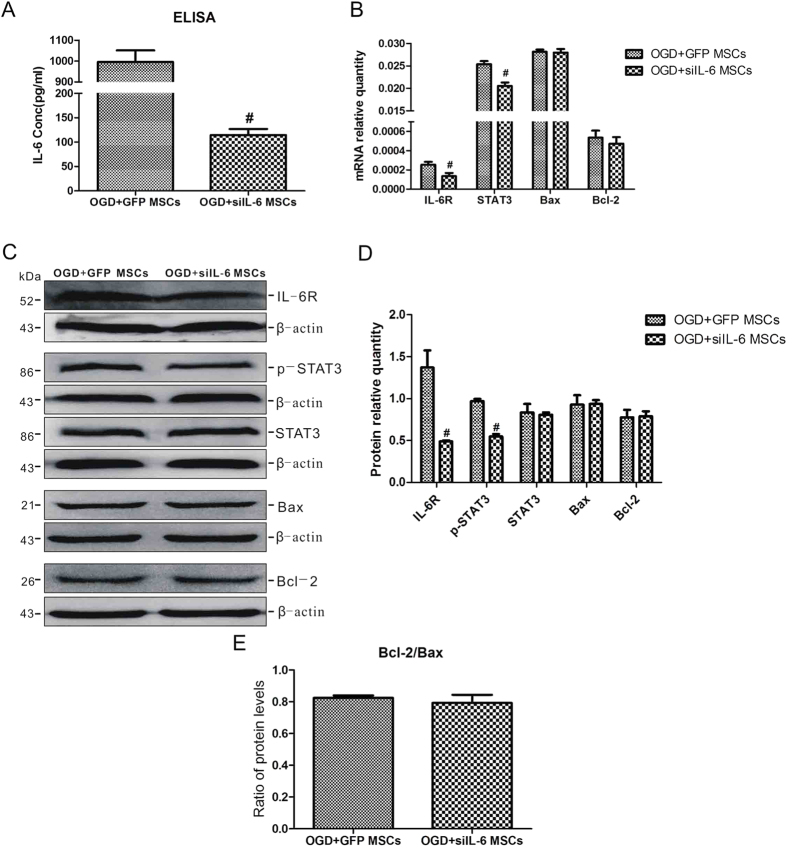
Silencing of IL-6 in MSCs suppresses the activation of the IL-6/STAT3 signalling pathway but has no effect on the ratio of Bcl-2/Bax in OGD-injured neurons. (**A**) The IL-6 concentration in the culture medium of OGD-injured neurons co-cultured with OGD + GFP MSCs and OGD + siIL-6 MSCs. n = 5. (**B**) The mRNA expression levels of IL-6R, STAT3, Bax and Bcl-2 in the two groups. n = 4. (**C**) Representative western blot of IL-6R, p-STAT3, STAT3, Bax and Bcl-2 expression in neurons in the two groups. n = 4. (**D**) The quantifications of WB signal in C. (**E**) The ratio of Bcl-2 to Bax protein levels in the two groups. The results are presented as the mean ± SEM. ^#^*P* < 0.05 vs. the OGD + GFP MSCs group.

We further validated this result in OGD-injured neurons co-cultured with siIL-6 MSCs and GFP MSCs. As shown in Fig. 5A, the levels of IL-6 released from OGD-injured neurons co-cultured with siIL-6 MSCs was significantly lower than that in the OGD + MSCs group, demonstrating that siRNA targeting the IL-6 gene in MSCs markedly reduced the level of IL-6 expression in OGD-injured neurons. The suppression of IL-6 expression also resulted in significant decreases in the mRNA and protein levels of IL-6R and STAT3 (Fig. 5B–D). However, the mRNA and protein expression levels of Bax and Bcl-2 were not influenced by the changes in IL-6 expression (Fig. 5B–D). The ratio of Bcl-2 and Bax protein expression levels did not differ between the two groups (Fig. 5D,E). Taken together, these data indicate that IL-6 release by MSCs activates the IL-6/STAT3 signaling pathway in neurons following OGD but has no effect on the suppression of apoptosis.

### MSCs influence the ratio of Bcl-2/Bax in OGD-injured astrocytes via the IL-6/STAT3 signaling pathway

**Figure 6:**
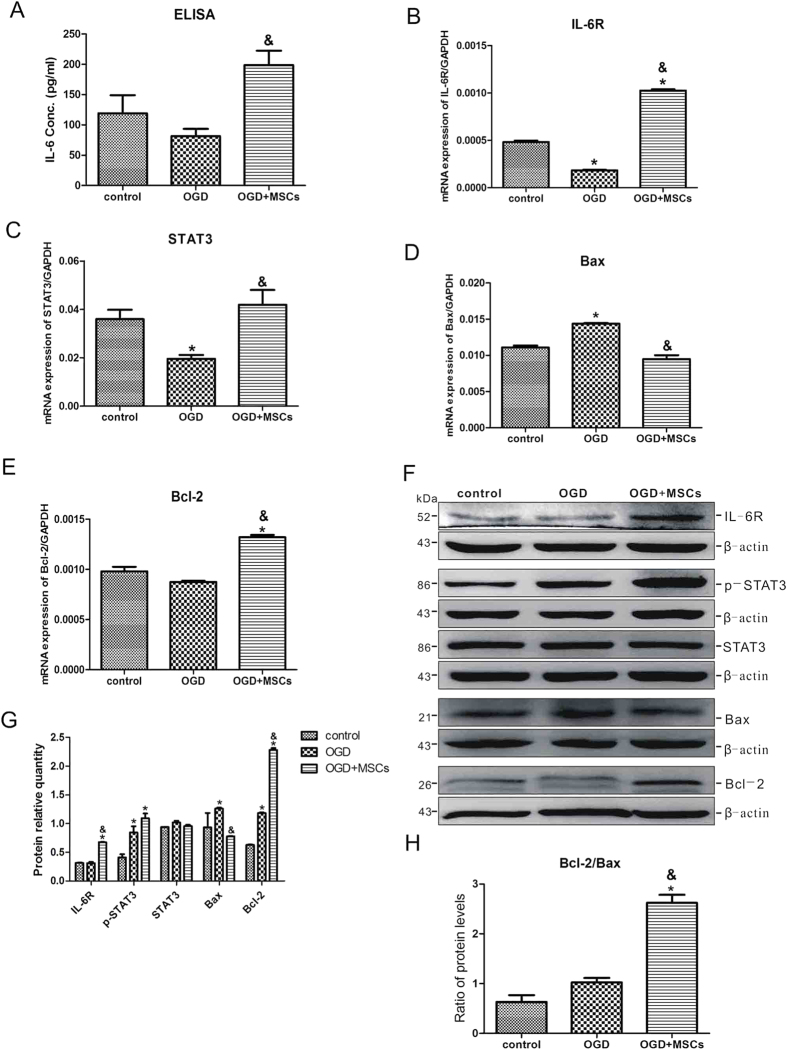
MSC co-culture activates the IL-6/STAT3 signalling pathway and suppresses apoptosis in OGD-injured astrocytes. (**A**) The concentration of IL-6 released in the culture medium of normal astrocytes and OGD-injured astrocytes co-cultured with or without MSCs. n = 5. (**B–E**) The mRNA expression levels of IL-6R, STAT3, Bax and Bcl-2 in the three groups. n = 4. (**F**) Representative western blot of IL-6R, p-STAT3, STAT3, Bax and Bcl-2 expression in astrocytes in the three groups. n = 4. (**G**) The quantifications of WB signal in F. (**H**) The ratio of Bcl-2 to Bax protein levels among the three groups. The results are presented as the mean ± SEM. *P < 0.05 vs. the control group, ^&^*P* < 0.05 vs. the OGD group.

We next shifted our attention to astrocytes, the most abundant neuroglial cell type in the central nervous system^21^. As shown in Fig. 6A, OGD injury downregulated the release of IL-6 in astrocytes, whereas MSC co-culturing significantly upregulated the expression of IL-6 in OGD-injured astrocytes. The changes in the mRNA expression levels of IL-6R, STAT3 and Bcl-2 completely mirrored that of IL-6 (Fig. 6B,C,E). However, the mRNA expression level of Bax in astrocytes was increased by OGD injury compared with the control group, whereas MSC co-culture suppressed the mRNA expression level of Bax in OGD-injured astrocytes (Fig. 6D). The protein expression levels of IL-6R, STAT3 and Bcl-2 were slightly upregulated in the OGD group and significantly increased in the OGD + MSCs group. The protein expression level of Bax was similar to its mRNA expression level (Fig. 6F,G). The ratio of Bcl-2/Bax protein levels demonstrated that MSC co-culture significantly increased the protein expression of Bcl-2 in OGD-injured astrocytes (Fig. 6H). Based on these data, we speculated that MSCs play an important role in anti-apoptosis of OGD-injured astrocytes by activating the IL-6/STAT3 signaling pathway.

**Figure 7:**
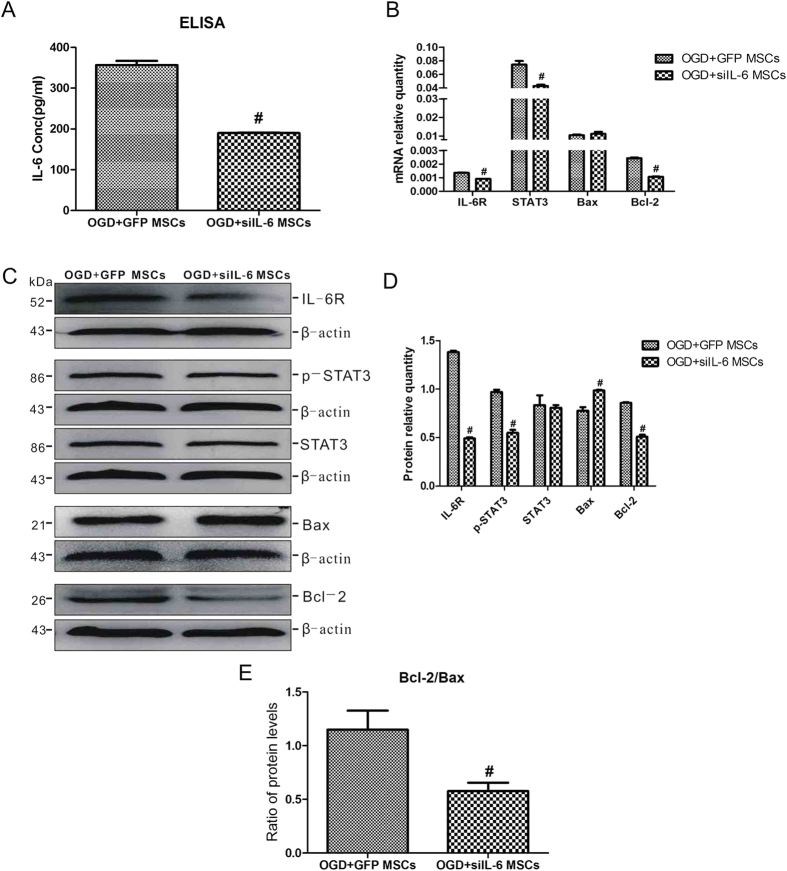
siIL-6 MSC co-culture impairs the activity of the IL-6/STAT3 signalling pathway and suppresses anti-apoptosis in OGD-injured astrocytes. (**A**) The concentrations of IL-6 in the culture medium of astrocytes co-cultured with OGD + GFP MSCs or OGD + siIL-6 MSCs as determined by ELISA. n = 5. (**B**) The mRNA expression levels of IL-6R, STAT3, Bax and Bcl-2 in the two groups. n = 4. (**C**) Representative western blot of IL-6R, p-STAT3, STAT3, Bax and Bcl-2 expression in the two groups. n = 4. (**D**) The quantifications of WB signal in C. (**E**) The ratio between Bcl-2 and Bax protein levels in the two groups. The results are presented as the mean ± SEM. ^#^*P* < 0.05 vs. the OGD + GFP MSCs group.

To verify this speculation, siIL-6 MSCs and GFP MSCs were co-cultured with OGD-injured astrocytes. As shown in Fig. 7A, with the decrease in IL-6 levels in OGD-injured astrocytes following co-culture with siIL-6 MSCs, the expression levels of IL-6R, STAT3 and Bcl-2 all decreased at both the mRNA and protein levels. However, opposite changes in the protein expression level of Bax were observed though the mRNA level of Bax didn’t differ between the two groups (Fig. 7B–D). The ratio of Bcl-2/Bax protein levels also revealed that the expression level of Bcl-2 was significantly suppressed in the OGD + siIL-6 MSCs group (Fig. 7D,E). These findings further indicate that IL-6 released by MSCs is a key factor in anti-apoptosis of OGD-injured astrocytes via activation of the IL-6/STAT3 signaling pathway.

### MSCs suppress apoptosis in OGD-injured astrocytes instead of OGD-injured neurons by releasing IL-6

**Figure 8:**
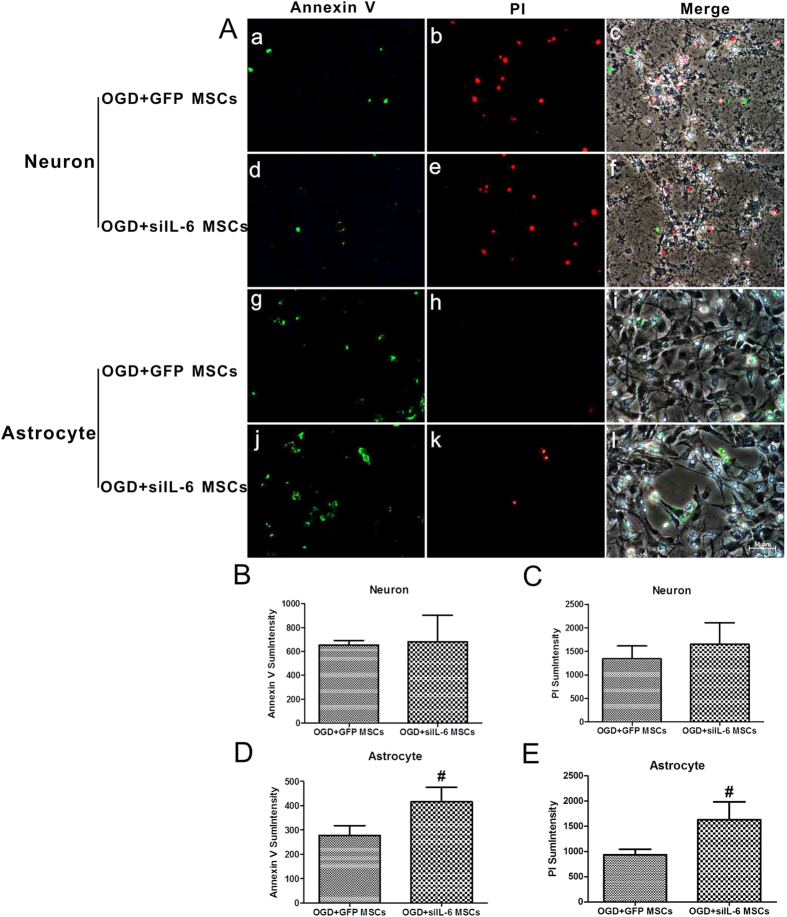
siIL-6 MSC co-culture suppresses anti-apoptosis of OGD-injured astrocytes but has no effect on neurons following OGD injury. (**A**) Annexin V-FITC/PI double staining of neurons or astrocytes in the OGD + GFP and OGD + siIL-6 MSCs groups. a, d, g and j. Annexin-V-positive cells in neurons or astrocytes in the OGD + GFP group or OGD + siIL-6 MSCs group. b, e, h and k. PI-positive cells in neurons or astrocytes in the OGD + GFP group or OGD + siIL-6 MSCs group. c, f, i and l. Annexin-V-positive cells and PI-positive cells merged with bright-field images of neurons or astrocytes in the OGD + GFP group or OGD + siIL-6 MSCs group. n = 5. (**B,C**) Annexin-V and PI sum intensity of neurons in the OGD + GFP and OGD + siIL-6 MSCs groups. (**D,E**) Annexin-V and PI sum intensity of astrocytes in the OGD + GFP and OGD + siIL-6 MSCs groups. Scale bar = 50 μm. The results are presented as the mean ± SEM. ^#^*P* < 0.05 vs. OGD + GFP MSCs.

To confirm that the target cells of IL-6 released from MSCs are astrocytes rather than neurons, Annexin V-FITC/PI double staining was used to detect apoptosis of neurons and astrocytes in both the OGD + GFP MSCs and OGD + siIL-6 MSCs groups. As shown in Fig. 8A–C, there were no differences in Annexin V-positive neurons and PI-positive neurons between the OGD + GFP MSCs group and OGD + siIL-6 MSCs group, indicating that the endogenous IL-6 of MSCs did not affect neuron apoptosis following OGD injury. However, the levels of both Annexin V-positive astrocytes and PI-positive astrocytes were both significantly higher in the OGD + siIL-6 MSCs group than the OGD + GFP MSCs group (Fig. 8A,D,E), further demonstrating that the MSCs suppressed apoptosis in OGD-injured astrocytes via IL-6 release. These results provide strong evidence supporting previous conclusions that IL-6 is the key factor by which MSCs exert an anti-apoptotic role in astrocytes following HIBD.

### IL-6 release from MSCs reduces apoptosis in the brains of HIBD rats by increasing Bcl-2 expression levels in astrocytes

**Figure 9:**
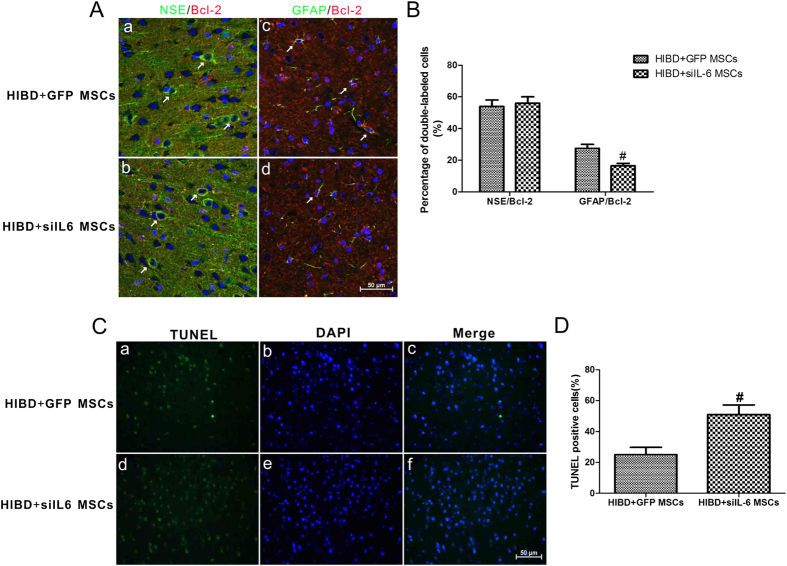
siIL-6 MSCs transplantation reduces Bcl-2 expression levels in the astrocytes to suppress the anti-apoptotic effect of MSCs but has no effect on neurons following OGD treatment. (**A**) Double immunofluorescence staining of NSE or GFAP together with Bcl-2 in the cerebral cortex of rats in the HIBD + GFP MSCs and HIBD + siIL-6 MSCs groups. a and b. Double immunofluorescence staining of NSE together with Bcl-2. The white arrows indicate co-localisation of NSE and Bcl-2. c and d. Double immunofluorescence staining of GFAP together with Bcl-2. The white arrows indicate co-localisation of GFAP and Bcl-2. n = 5. (**B**) Quantification of the percentage of NSE/Bcl2 or GFAP/Bcl2 double-labelled cells as presented in A. (**C**) TUNEL staining of the rat cerebral cortex of the HIBD + GFP group and HIBD + siIL-6 MSCs group. a and d. TUNEL-positive cells. b and e. DAPI-stained cells. c and f. Merged images of TUNEL-positive cells and DAPI-stained cells. n = 6. (**D**) The percentage of TUNEL-positive cells in the HIBD + GFP and HIBD + siIL-6 MSCs groups. Scale bar = 50 μm. The results are presented as the mean ± SEM. ^#^*P* < 0.05 vs. HIBD + GFP MSCs.

To further verify the above *in vitro* results, we conducted double immunofluorescence staining and TUNEL staining with the brains of rats in the HIBD + GFP MSCs and HIBD + siIL-6 MSCs groups. No difference in double immunofluorescence staining of NSE and Bcl-2 was observed between the two groups (Fig. 9A-a,A-b,B), whereas immunofluorescence staining of GFAP together with Bcl-2 revealed reduced co-localization of Bcl-2 with GFAP in the HIBD + siIL-6 MSCs group compared with the HIBD + GFP MSCs group (Fig. 9A-c, A-d, B). TUNEL staining demonstrated that the amount of TUNEL-positive cells was significantly greater in the HIBD + siIL-6 MSCs group than the HIBD + GFP MSCs group (Fig. 9C,D). These data further demonstrated that the target cells of IL-6 following transplantation of MSCs into the brains of HIBD rats are astrocytes and that IL-6 plays an anti-apoptotic role by increasing the expression level of Bcl-2.

## Discussion

This study addressed whether IL-6 is the key factor in alleviating the impairment of learning and memory function in HIBD rats following MSC transplantation and identified the target cells of IL-6. We demonstrated that the neuroprotective effect of MSCs in HIBD rats is mediated by endogenous IL-6 and that IL-6 mainly acts on astrocytes to play a role in anti-apoptosis via the IL-6/STAT3 signaling pathway.

MSCs are an important source of multipotent cells used in cell-based therapy. Increasing evidence indicates that MSC transplantation attenuates neurological lesions caused by brain injury^3^,^4^,^5^,^6^,^22^,^23^. The potential mechanisms of MSC-induced neurorestorative effects after brain injury include cell replacement, trophic support from transplanted cells, immunomodulation and stimulation of endogenous brain repair processes. The therapeutic effect of MSCs was initially ascribed to their potential to differentiate into neurons and replace the injured neurons in the pathological area^24^,^25^ (34, 35). However, only approximately 20% of the transplanted MSCs differentiated into neurons expressing neuronal markers. In addition, expression of neuronal markers did not indicate owning neuronal function^26^. Newly formed neurons died if they did not receive appropriate trophic signals or establish functional synaptic connections^27^. Therefore, subsequent studies focused on the immunological function of MSCs in lesion sites. Crigler *et al*. demonstrated that MSCs transplanted at sites of injury promoted functional recovery by producing trophic factors that induce the survival and regeneration of host neurons^28^. Van Velthoven *et al*. observed that MSC transplantation stimulated HI-induced neural progenitor cell proliferation in the lesion hemisphere and enhanced survival of proliferating cells^4^. The immunomodulatory function of MSCs attracted more attention among all studies^5^,^29^. Our findings add strong evidence for the immunomodulatory function of MSCs because the *in vivo* data suggested that the neuroprotective effect of MSC transplantation in HIBD rats is mainly attributable to endogenous IL-6 released by MSCs.

IL-6 was originally identified as a T cell-derived lymphokine that induces final maturation of B cells into antibody-producing cells. The biological function of IL-6 is not restricted to the immune system and is also involved in hematopoiesis, acute phage reactions and neural systems^30^. In the normal brain, IL-6 participates in the modulation of information processing because it potentiates the release of the neurotransmitter gamma-amino butyric acid (GABA)^31^. IL-6 also plays substantial roles in maintaining homeostasis in the brain by directing neurogenesis, astrogliosis, microgliosis and controlling blood–brain barrier integrity, which could be upregulated by various brain injuries^14^. An increase in serum IL-6 levels has been reported in both adult ischemic stroke and neonatal hypoxic-ischemic encephalopathy^32^,^33^. The role of IL-6 in the injured brain is controversial. Huang *et al*. speculated that upregulated IL-6 might act on the vascular endothelium by increasing harmful mediators and mediating inflammatory cascades that to lead to the exacerbation of cerebral ischemic damage^34^. However, IL-6 promotes post-traumatic healing in the CNS by enhancing angiogenesis^35^. The results of our study support the neuroprotective effects of IL-6 because the ability of MSCs to improve learning-memory function in HIBD was reduced when IL-6 expression was blocked.

The most basic classification of cells in the CNS is the distinction between neurons and glia cells. Neurons are the main players in neural information processing because they have the unique capacity to generate action potentials and communication via chemical synapses. Glia cells are usually divided into oligodendrocytes, microglia and astrocytes based on differences in morphology. The function of oligodendrocytes is to participate in myelination. Microglia are mainly implicated in phagocytosis and defences. Astrocytes are the more abundant cells in the CNS and generally outnumber neurons by more than fivefold. Astrocytes play multiple roles in the brain, including controlling the formation, maintenance, function, and removal of neuronal synapses, helping to control blood flow and regulating the blood-brain barrier together with pericytes^36^,^37^. Based on the importance and quantity of neurons and astrocytes, we chose these cell types as subjects in our study to explore the target cells of endogenous IL-6 released by MSCs. Our findings demonstrated that the target cells of IL-6 from MSCs were astrocytes and not neurons. The *in vitro* study demonstrated that MSC co-culture increased the expression levels of anti-apoptotic protein Bcl-2 and decreased the expression levels of pro-apoptotic protein Bax simultaneously in OGD-injured astrocytes. However, no differences in Bcl-2 and Bax protein levels were observed in OGD-injured neurons with or without MSC treatment. Although several studies have emphasised that reactive astrogliosis after CNS injury prompts glial scar formation, which inhibits axon regeneration and impedes functional recovery^38^,^39^, more studies have indicated a neuroprotective role of astrocytes in CNS injury^40^,^41^,^42^. Even astrocytes are generally more resilient than neurons after injury, and severe damage leads to astrocyte dysfunction and, consequently, increased neuronal death^43^. Co-culture with MSCs may protect astrocytes from apoptosis and, consequently, prevent further neuronal damage. However, the exact biological function of the actions of MSCs on neurons requires further study.

IL-6 has multiple biological functions through different signaling pathways. Before activating the downstream pathway, IL-6 must combine with its receptor IL-6R and glycoprotein 130 (gp130). The signal-transducing subunit gp130 is ubiquitously present, whereas IL-6R expression is predominantly restricted to hepatocytes, neutrophils, monocytes and macrophages. Other cells that do not express IL-6R respond to IL-6 in the presence of soluble IL-6R (sIL-6R)^44^. These complexes activate JAKs, which subsequently phosphorylate STAT proteins. This signalling pathway is referred to as the JAK–STAT pathway. Among the seven STAT isoforms, only STAT-3 participates in IL-6 signaling^12^. Another major signaling cascade that is activated by IL-6 is the MAPK pathway, which is also activated by JAKs. A third signaling cascade activated by IL-6 is the PI3K/Akt pathway^13^. The majority of studies have indicated that IL-6 signaling in the CNS is mediated by STAT-3^15^. Thus, we focused on the IL-6/STAT3 signaling pathway in the present study. Our data suggested that endogenous IL-6 release by MSCs upregulated the levels of IL-6R and p-STAT3 in OGD-injured astrocytes. In addition, the ratio between Bcl-2 and Bax, which are the downstream factors of the STAT3 signaling pathway, was significantly increased. Consistent with the findings of other studies, these results indicated that IL-6 inhibits the apoptosis of astrocytes by activating the IL-6/STAT3 signaling pathway^45^,^46^. Subsequent studies should focus on the exact mechanisms of anti-apoptosis in astrocytes following MSC co-culture.

In conclusion, we demonstrated that endogenous IL-6 release from transplanted MSCs exerts remarkable neuroprotective effects on improving learning and memory function in rats subjected to HIBD. This protective function of IL-6 targets astrocytes of the CNS to inhibit apoptosis via the IL-6/STAT3 signaling pathway. The novel information provided by this study may facilitate the application of MSCs in treating HIBD.

## Methods

### Animal groups

All animal experimental protocols were approved by the Animal Experimentation Ethical Committee of the Zoology Center at Chongqing Medical University (Chongqing, China) and were carried out in accordance with the approved guidelines. Specific pathogen-free (SPF)-grade Sprague-Dawley (SD) rats (8 weeks old) were purchased and housed in the Experimental Animal Center of Chongqing Medical University [SCXK (Yu) 2012-0001]. Food and water were available *ad libitum*. Seven-day postnatal rats were used to establish the HIBD model as reported previously^47^. Briefly, left carotid artery ligation was performed on 7-day-postnatal rats. Two hours later, the pups were exposed to 8% oxygen at 37 °C for 2.5 hours. Then, they were returned to their dams.

All pups were randomly assigned to one of the following five groups: sham, HIBD, HIBD + MSCs, HIBD + GFP MSCs and HIBD + siIL-6 MSCs. The rats in the sham group were only subjected to a cervical skin incision that was subsequently sutured. The HIBD rats of the HIBD + MSCs, HIBD + siIL-6 MSCs and HIBD + GFP MSCs groups were intracerebroventricularly injected with 2 × 10^5^ MSCs subjected to various different treatments, as described below, in 5 μL of phosphate-buffer saline (PBS) (Hyclone, USA) at 5 days post-hypoxia-ischaemia^48^. The pups in the HIBD group were injected with 5 μL of PBS as a control.

### Morris water maze

Five weeks after HIBD, 15 rats per group were subjected to the Morris water maze task (MWM SLY-WMS 2.0, China) to evaluate their spatial learning and memory functions, as previously described^49^. Briefly, on the first day of training, the rats were exposed to a visible platform to assess their visual capability. On the second through fifth days, the rats were continuously trained using the invisible platform to enhance their learning and memory function. The average escape latency and path length to locate the platform were recorded from the first to the fifth day. On the sixth day, a probe trial was performed without an escape platform, and the number of times that the rats crossed the former platform location in 60 s was recorded.

### Object-in-place task

Five-week-old rats in each group were used to conduct the object-in-place task. The animals were tested in a 1-m^3^ open-topped arena made of black Plexiglas. This task comprised a sample phase and a test phase separated by a 5-min delay^50^. Before the task, the subjects experienced a pre-training phase, during which rats were placed in the empty arena to adapt to the environment for 10 min for 2 consecutive days. During the sample phase, the rats were placed in the centre of the arena, which contained 4 different toys in the corners. Each rat was allowed to explore the toys for 5 min. During the delay period, the toys were cleaned with alcohol to remove olfactory cues and any sawdust that had adhered to the toys, and the positions of two adjacent toys were exchanged. During the test phase, the rats were returned to the arena and were allowed to investigate the toys for 3 min. The ratio of the time spent investigating the two toys whose position had been changed to the time spent exploring the two toys whose positions were not changed was calculated.

### Long-term potentiation (LTP) recording

Two-month-old rats in each group were used in this experiment, which was performed as described previously^51^. The fEPSPs in hippocampal slices were acquired using a Multi-clamp 700B amplifier and the pClamp 10 software package (Molecular Devices, USA). A 20-min baseline was recorded every 2 min at an intensity that evoked a response of 50% of the maximum evoked response. LTP was induced by HFS at 100 Hz for 1 s and was recorded every 2 min for 60 min after induction.

### Preparation of MSCs and treatment

Rat primary MSCs were isolated and expanded as described previously^52^. A rat siIL-6-transduced recombinant lentivirus was constructed (NeuronBiotech Co., Ltd., China) and was used to infect MSCs. Lines of siIL-6 MSCs were screened using puromycin (AMRESO, USA) as previously described^53^. A vector expressing green fluorescent protein (GFP) served as a negative control (GFP MSCs).

### Preparation and treatment of primary hippocampal neurons

Primary culture of hippocampal neurons was prepared from embryonic day 17–18 (E 17–18) SD rats as described previously^54^. The hippocampal neurons were cultured for 5 d and then subjected to OGD injury. Briefly, the cells were incubated in Earle's balanced salt solution (EBSS; Hyclone, USA) and cultured in an incubator containing 5% O_2_/5% CO_2_ (Thermo, USA) for 1.5 h; then, EBSS was replaced with standard neuronal culture medium. Cells cultured in standard neuronal culture medium in the presence of ambient (16%) O_2_/5% CO_2_ served as controls. Immediately upon the end of OGD treatment, the injured neurons were treated with either (1) neural basal medium as a control or (2) a separated co-culture with MSCs subjected to different treatments, as described above, in a transwell insert (Millipore, USA). Briefly, MSCs subjected to different treatments were cultured in the upper chamber of the transwell insert of the six well plates in which the neurons were cultured. After 24 h, total neuronal RNA was isolated and purified using an RNA extraction kit (BioTeke, China). After 48 h, the culture medium was collected from each group, and total protein was extracted using an extraction kit (BioTeke, China).

### Preparation and treatment of primary astrocytes

Rat cortical astrocytes were obtained as previously described^55^. Primary astrocytes were prepared from one-day-old SD rats. Immunofluorescence staining for glial fibrillary acidic protein (GFAP) indicated that 95% of the cells were astrocytes. Astrocytes were subjected to the same treatments as hippocampal neurons.

### Enzyme-linked immunosorbent assay (ELISA)

The levels of IL-6, IL-1β, IL-8, IL-10, TNFα and IFNβ in the culture medium of primary MSCs were detected using ELISA kits from Beijing 4A Biotech Co., Ltd. (China). The left hippocampus was collected from the rats in the HIBD + GFP MSCs and HIBD + siIL-6 MSC groups 7 d or 14 d after HIBD, and the levels of IL-6 in the hippocampal tissue of the two groups were measured using an ELISA kit (Raybiotech, Inc., USA). The levels of IL-6 in the culture medium collected from neurons and astrocytes in various treatment groups following OGD were detected using an ELISA kit (Beijing 4A Biotech Co., Ltd., China). The colorimetric optical density was detected at a wavelength of 450 nm. The cytokine levels were calculated based on a standard curve constructed for each assay, and each assay was performed in triplicate.

### Western blotting

Total protein extracted from primary neurons or astrocytes was used for western blotting. The membranes were incubated in primary antibodies including anti-IL6R (1:400, Abcam, USA), anti-STAT3 (1:800, ABZOOM, USA), anti-Bax (1:200, Sigma, USA), anti-Bcl-2 (1:800, Sigma, USA) and anti-β-actin (1:500, Santa Cruz, USA) at 4 °C overnight, followed by incubation in HRP-conjugated secondary antibodies (Santa Cruz, USA) at room temperature for 1 h. Then, the protein bands were detected using a chemiluminescent HRP substrate (Millipore, USA). The images were captured using a Syngene GBox Imaging System.

### Real-time PCR

Total RNA isolated from primary neurons or astrocytes was used for real-time PCR. A 500-ng sample of total RNA was reverse-transcribed into cDNA using the PrimeScript® RT reagent kit (Takara, Japan) according to the manufacturer’s protocol. The primer sequences for rat IL-6, IL-6R, STAT3, Bax, Bcl-2 and GAPDH are provided in Table 1. The genes of interest were amplified via real-time PCR using a CFX Connect Real-Time PCR thermocycler (BioRad, USA) and SYBR II Premix Ex Taq^TM^ (Takara, Japan). PCR was performed according to the following protocol: 95 °C for 30 s followed by 40 cycles of 95 °C for 5 s and 60 °C for 30 s. The melting curve was analysed, and a specific standard curve was generated for each gene in parallel. Each sample was quantified in triplicate. The gene expression results were calculated by normalising the concentration of the target gene to that of the endogenous levels of GAPDH using CFX Manager Software.

### Cell apoptosis assay

An Annexin V-FITC/PI double-staining kit (KeyGen Biotech, China) was used to detect apoptosis of neurons and astrocytes in the OGD + GFP MSC and OGD + siIL-6 MSC groups according to the manufacturer’s instructions. Briefly, after treatment, the cells in the two groups were washed three times with PBS and then incubated in 5 μl of FITC-conjugated Annexin V and 5 μl of PI in 100 μl of binding buffer for 5 min at room temperature. Apoptotic changes to neurons and astrocytes were observed under an inverted fluorescence microscope (Nikon, Japan) and photographed using a digital camera (Nikon, Japan).

### TUNEL staining and double immunofluorescence staining assays

TUNEL staining and double immunofluorescence staining for Bcl-2 and either NSE or GFAP were performed on the brains of rats subjected to HIBD following treatment with GFP MSCs or siIL-6 MSCs. Briefly, all rats were anaesthetised and transcardially perfused with 4% paraformaldehyde (Kelong Chemical Co., Ltd., China). Slices of brain tissue with a thickness of 12 μm were sectioned using a Leica CM3050 S cryostat. DNA fragmentation of apoptotic cells was detected using a TUNEL kit (KeyGen Biotech, China) according to the manufacturer’s instructions. The TUNEL-positive cells were counted from four randomly selected fields in each group, and the cell counts were expressed as a percentage of the total number of cells.

The sections used for double immunofluorescence staining were incubated in goat anti-Bcl-2 (1:100, Abcam, USA) together with either rabbit anti-NSE (1:100, Abcam, USA) or mouse anti-GFAP (1:100, Abcam, USA) overnight at 4 °C. The primary antibodies were visualised using Alexa Fluor 488-conjugated chicken anti-rabbit (or anti-mouse) IgG (1:100, Life Technologies, USA) and Alexa Fluor 594-conjugated donkey anti-goat IgG (1:100, Jackson, USA), and 4’,6-diamidino-2-phenylindole (DAPI, 1:100, Yeasen, China) was used to stain nuclei. The images were captured using a Nikon laser confocal microscope (Nikon, Japan).

### Statistical analyses

All data are expressed as means ± standard error of the mean (mean ± SEM) and were analysed using GraphPad Prism 5.0 software (GraphPad Software, Inc., USA). Significant differences between groups were determined via one-way repeated-measures analysis of variance (ANOVA) followed by Duncan’s multiple range tests. Significance was set at *P* < 0.05.

## Additional Information

**How to cite this article**: Gu, Y. *et al.* Endogenous IL-6 of mesenchymal stem cell improves behavioral outcome of hypoxic-ischemic brain damage neonatal rats by supressing apoptosis in astrocyte. *Sci. Rep.* **6**, 18587; doi: 10.1038/srep18587 (2016).

## Figures and Tables

**Table 1:** Primers used for real-time PCR of target genes.

Primers	Sequences
**IL-6**	F: 5′-ACAGCCACTGCCTTCCCTAC-3′
	R: 5′-TTGCCATTGCACAACTCTTTTC-3′
**IL-6R**	F: 5′-TGAGAAGGAAAGCAAGACG-3′
	R: 5′-TGTTATGGGACCCTGATG-3′
**STAT3**	F: 5′-CCGCACTTTAGATTCATTG-3′
	R: 5′-TCACATCGGGGAGGTAGCA-3′
**Bax**	F: 5′-AAGTAGAAGAGGGCAACCAC-3′
	R: 5′-GATGGCAACTTCAACTGGG-3′
**Bcl-2**	F: 5′-CGGGAGAACAGGGTATGA-3′
	R: 5′-CAGGCTGGAAGGAGAAGAT-3′
**GAPDH**	F: 5′-CCTGGAGAAACCTGCCAAG-3′
	R: 5′-CACAGGAGACAACCTGGTCC-3′
